# The Effects of Genetic and Epigenetic Alterations of BARD1 on the Development of Non-Breast and Non-Gynecological Cancers

**DOI:** 10.3390/genes11070829

**Published:** 2020-07-21

**Authors:** Andrea K. Watters, Emily S. Seltzer, Danny MacKenzie, Melody Young, Jonathan Muratori, Rama Hussein, Andrej M. Sodoma, Julie To, Manrose Singh, Dong Zhang

**Affiliations:** Department of Biomedical Sciences, College of Osteopathic Medicine, New York Institute of Technology, Old Westbury, New York, NY 11568, USA; awatters@nyit.edu (A.K.W.); eseltz01@nyit.edu (E.S.S.); dmackenz@nyit.edu (D.M.J.); myoung08@nyit.edu (M.Y.); jmurator@nyit.edu (J.M.); rhusse04@nyit.edu (R.H.); asodoma@nyit.edu (A.M.S.); jto01@nyit.edu (J.T.); msingh74@nyit.edu (M.S.)

**Keywords:** BARD1, cancers, single nucleotide polymorphism (SNP), BARD1 isoforms

## Abstract

Breast Cancer 1 (*BRCA1*) gene is a well-characterized tumor suppressor gene, mutations of which are primarily found in women with breast and ovarian cancers. BRCA1-associated RING domain 1 (*BARD1*) gene has also been identified as an important tumor suppressor gene in breast, ovarian, and uterine cancers. Underscoring the functional significance of the BRCA1 and BARD1 interactions, prevalent mutations in the *BRCA1* gene are found in its RING domain, through which it binds the RING domain of BARD1. BARD1-BRCA1 heterodimer plays a crucial role in a variety of DNA damage response (DDR) pathways, including DNA damage checkpoint and homologous recombination (HR). However, many mutations in both BARD1 and BRCA1 also exist in other domains that significantly affect their biological functions. Intriguingly, recent genome-wide studies have identified various single nucleotide polymorphisms (SNPs), genetic alterations, and epigenetic modifications in or near the B*ARD1* gene that manifested profound effects on tumorigenesis in a variety of non-breast and non-gynecological cancers. In this review, we will briefly discuss the molecular functions of BARD1, including its BRCA1-dependent as well as BRCA1-independent functions. We will then focus on evaluating the common BARD1 related SNPs as well as genetic and epigenetic changes that occur in the non-BRCA1-dominant cancers, including neuroblastoma, lung, and gastrointestinal cancers. Furthermore, the pro- and anti-tumorigenic functions of different SNPs and BARD1 variants will also be discussed.

## 1. Introduction

**Figure 1:**
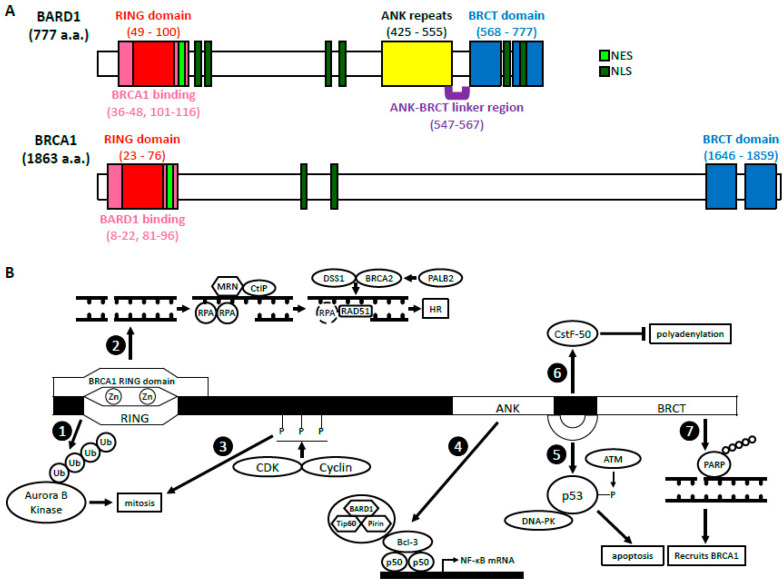
The structure and functions of BARD1 (**A**) BARD1 and BRCA1 domain structures. BARD1 and BRCA1 have similar RING domains located at their N-termini, BRCT domains located at their C-termini, as well as NES and NLS. The ANK repeats and the ANK-BRCT linker region are unique to BARD1. (**B**) A summary of the biological functions of BARD1. BRCA1-dependent: The BARD1 RING domain interacts with the RING domain of BRCA1. (1) The E3 ligase activity of BARD1-BRCA1. (2) The HR function of BARD1-BRCA1. BRCA1-independent: (3) CDK2-CyclinA1/E1 and CDK1-CyclinB phosphorylate BARD1, which then facilitates mitotic progression. (4) BARD1 forms a complex with Tip60 and Pirin that interacts with p50 and Bcl-3 through BARD1’s ANK and BRCT domains. This complex binds the NF-κB promoter and regulates its transcription. (5) BARD1, together with DNA-PK, stabilizes p53 through its ANK-BRCT linker region, which allows phosphorylation by ATM and induction of apoptosis. (6) The ANK-BRCT linker region also binds CstF-50 and together they prevent polyadenylation of mRNA in response to DNA damage. (7) BARD1 binds the poly (ADP-ribose) through its BRCT domain and recruits BRCA1 to the DNA damage sites.

BARD1 was discovered in 1996 by Baer’s group who sought out to identify proteins that interacted with the N-terminus of BRCA1 through a two-hybrid screening [1]. Of the 16 candidates identified, only BARD1 was able to directly interact with and form a stable complex with BRCA1 in mammalian cells through its RING domain, hence its name, BRCA1-associated RING domain 1 (*BARD1*) gene. *BARD1* gene is localized to 2q35 and encodes a full-length (FL) protein (BARD1-FL) composed of 777 amino acids (a.a.) [1,2,3]. There are many structural similarities between BRCA1 and BARD1 (Figure 1A). The RING domain of BARD1 consists of a.a. 49–100 and is rich in cysteine and histidine, which bind to two zinc ions [1,4]. However, the RING domain of BARD1 is slightly shorter and lacks the central α-helix as well as the third β-pleated sheet when compared to that of BRCA1. It is flanked by two α-helices (a.a. 36–48 and 101–116), which interact with the helices adjacent to the RING domain of BRCA1 to form a heterodimer. Similar to BRCA1, BARD1 also contains two tandem copies of the BRCA1 C-terminal (BRCT) domain located at a.a. 568–777 [5,6]. The domain is composed of BRCT1 (a.a. 568–654) connected to BRCT2 (a.a. 669–777) by an α-helix. BRCT1 has a hydrophilic binding pocket, which binds to phospho-peptides, just like BRCA1. The second binding pocket is located between the two BRCT domains and is relatively hydrophobic. The amino acid sequences of the second binding pocket of BARD1 are different from those in BRCA1, suggesting unique ligand interactions. BARD1, like BRCA1, contains a nuclear export sequence (NES) at a.a. 102–120 that allows for its transport out of the nucleus and into the cytoplasm [7]. It also contains six nuclear localization signals (NLS) which allow the protein to move back into the nucleus [8]. The NLS sequences are located at a.a. 127–130, 139–155, 321–337, 365–371, 657–663, and 706–709.

BARD1’s structure differs from BRCA1 also due to the presence of ankyrin (ANK) repeats at a.a. 425–555 [6,9]. This domain consists of four ANK repeats that compose a hydrophobic helical core. The fourth ANK repeat differs in its sequence and is shortened but maintains an overall similar structure. The non-traditional orientation of the fourth ANK may be attributed to a span of 20 amino acids (a.a. 547–567), which include and extend beyond the C-terminal ANK repeat domain. This region, which has no secondary structure, is thus called the ANK-BRCT linker region. Overall, the structural domains of BARD1 are critical for their biological functions because they are vital for various protein-protein and protein-DNA interactions. 

**Figure 2:**
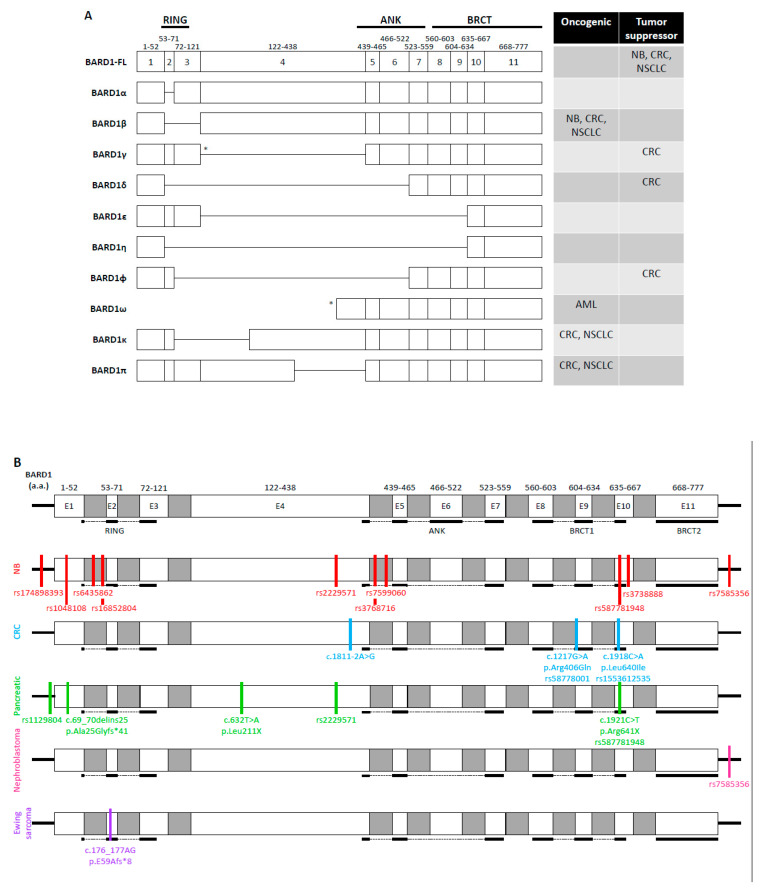
BARD1 variants in non-breast and non-gynecological cancers (**A**) BARD1 isoforms. Full-length BARD1 (BARD1-FL) and different variants that occur due to alternative splicing. The table depicts in which cancer types the isoforms are oncogenic or tumor suppressors. * indicates multiple forms of alternative splicing. The γ isoform is either composed of only exons 1–3 or exons 1–3, 5–11. The ω1 isoform is depicted in this figure, which begins at a.a. 382. The ω2 isoform begins in exon 4 at a.a. 407 while the ω3 isoform begins in exon 7 at a.a. 538. NB: neuroblastoma. CRC: colorectal cancer. NSCLC: non-small cell lung cancer. AML: acute myeloid leukemia. (**B**) SNPs and genetic mutations in the *BARD1* gene. BARD1 is composed of 11 exons (white) and introns (gray) and includes 5′ and 3′ UTR (black lines). Genetic variants of BARD1 in NB (red), CRC (blue), pancreatic cancer (green), nephroblastoma (pink), and Ewing sarcoma (purple) are depicted as lines localized to the region of the mutation. Only exons are drawn to scale.

Due to the pronounced role of BRCA1 and BRCA2 in hereditary breast and ovarian cancer, different mutations and variants of BARD1 were first investigated in breast cancers and various gynecological cancers in the late 1990s and early 2000s [10,11,12]. The genetic changes in *BARD1* include missense mutations, nonsense mutations, and deletions. For example, women with the missense mutation C557S, just before the BRCT1 domain of BARD1, have an increased susceptibility to breast cancer [11,12]. Many studies have also identified various isoforms of BARD1 that result from alternative splicing (Figure 2A). The α isoform removes exon 2, while the β isoform splices out exons 2 and 3 [13]. The γ isoform loses either exon 4 [13] or exons 4–11 [14]. The δ isoform lacks exons 2 through 6 [15,16], while the ε isoform removes exons 4 to 9 [13]. The η isoform has exons 2–9 removed, and the ϕ isoform lacks exons 3 through 6. The ω isoform lacks exons 1–3 and encodes proteins of different lengths due to different translation starting sites in exons 4 and 5 [17]. The most recently identified isoforms are π, which has the C-terminal portion of exon 4 deleted, and κ, which lacks exon 3 and the N-terminal portion of exon 4 [14,18]. The RING domain of BARD1 is mapped to exons 2 and 3 [19]. Its ANK repeats span from the end of exon 4 to exon 7 while its BRCT domain encompasses exons 8–11. It is thought that the different isoforms may play a role in tumorigenesis through the disrupting of BARD1’s important protein-protein interactions. These variations have been well-studied in hereditary breast and ovarian cancers, however, the presence and functional consequences of these alterations in other cancer types are still being investigated.

In this review, we will first highlight the important biological functions of BARD1, both BRCA1-dependent and BRCA1-independent (Figure 1B). We will then describe different variations of BARD1 present in non-breast and non-gynecological cancers, which are not driven by mutations in either BRCA1 or BRCA2 genes. Specifically, we will summarize the clinical manifestations of single nucleotide polymorphisms (SNPs) in the *BARD1* gene and the expression of BARD1 isoforms in neuroblastoma (NB), gastrointestinal cancers, non-small cell lung cancer (NSCLC), nephroblastoma, Ewing sarcoma, and acute myeloid leukemia (AML). We will then discuss how these genetic alterations affect the domain structures of BARD1 and the implications of these changes in BARD1-mediated biological interactions and processes, including tumorigenesis.

## 2. BRCA1-Dependent Function of BARD1

### 2.1. BARD1-BRCA1 E3 Ubiquitin Ligase Activity

BARD1 and BRCA1 interact via the RING domains located at their respective N-termini. This interaction allows for the formation of a heterodimer with E3 ubiquitin ligase activity [20,21]. As an ubiquitin ligase, BARD1-BRCA1 promotes ubiquitination of targeted proteins and their proteasomal degradation; however, its atypical K6 ubiquitin linkages, though poorly understood, are thought to act in signal transduction [22,23,24]. BARD1-BRCA1 can even auto-ubiquitinate to increase its own activity levels and stability [22,25]. The heterodimer formation may be vital for the stabilization of BARD1 and BRCA1 as the loss of one protein drastically decreases the amount of the other protein [21].

Many of the heterodimer’s roles relate to facilitating mitosis. For example, BARD1-BRCA1 has been shown to localize to and ubiquitinate centrosome proteins, particularly γ-tubulin and the γ-tubulin ring complex, to inhibit microtubule nucleation at the centrosomes [26,27,28,29]. Loss of BARD1-BRCA1 at this step results in the rapid accumulation of fragmented or extra centrosomes [27,29]. Intriguingly, BRCA1, likely with the help of BARD1, can also promote DNA damage-induced centrosome amplification, possibly as a defense mechanism in response to prolonged DNA damage [30]. BARD1-BRCA1 ubiquitination activity is also required for TPX2, a spindle fiber organizer, to properly aggregate at the spindle poles [31]. Together, these studies confer both centrosome-dependent and -independent functions in spindle apparatus assembly during metaphase and anaphase. During telophase and cytokinesis, BARD1-BRCA1 ubiquitinates Aurora B kinase, a chromosomal segregation kinase, which leads to its turnover [32]. BARD1-BRCA1′s action against Aurora B is thought to confine it to the mitotic contractile ring. Interestingly, this likely occurs via Aurora B binding to TACC1 at the midbody with protection by BARD1β [3,32,33].

Finally, BARD1-BRCA1 E3 ubiquitin ligase activity has also been implicated in nucleosome and chromatin modulation for DNA repair as well as for signal transduction of estrogen and progesterone [25,34,35,36,37,38].

### 2.2. BARD1-BRCA1 in Homologous Recombination

BRCA1-BARD1 has long been implicated in homologous recombination (HR). Although the heterodimer has many biological functions, its role in HR is perhaps the most thoroughly investigated and likely contributes the most to its tumor-suppressing functions. Therein, the BARD1-BRCA1 complex has been primarily implicated in DNA end resection and presynaptic complex formation.

#### 2.2.1. DNA End Resection

To commit to HR, two processes must occur. First, the 53BP1-RIF1-Shieldin complex, which aggregates to protect dsDNA ends after double-strand break (DSB) formation, needs to be removed [39,40]. The BARD1-BRCA1 heterodimer is thought to facilitate this process by ubiquitinating histone H2A. This enables SMARCAD1 recruitment, a chromatin remodeler, which facilitates the removal of the 53BP1-containing complex [36]. Second, the 5′ ends of DSBs are resected to generate 3′ overhangs of ssDNA. These overhangs then serve as docking sites for the assembly of pro-HR proteins and commit DNA repair to the HR pathway. CtIP binds the BRCT domain of BRCA1 as well as the MRE11-RAD50-NBS1 (MRN) complex [41,42]. Together with other nucleases, the MRN-CtIP complex processes the DSB ends and promotes HR [43]. BARD1-BRCA1 interacting with CtIP indicates a potential function in DNA end resection. In line with this, studies have demonstrated that BRCA1 improved resection speed [44]. However, in all, this process is not exclusively BRCA1-dependent [45,46].

#### 2.2.2. Presynaptic Complex Formation

The presynaptic complex, or the presynaptic filament, refers to the RAD51-ssDNA nucleoprotein filament that is formed at the 3′ ends of resected DNA, which then searches and invades the homologous strand and forms a D-loop [47]. Immediately after DNA end resection, Replication Protein A (RPA) binds to and protects the ssDNA until RAD51 is loaded onto the ssDNA to replace them [48]. RAD51, a DNA recombinase, is a key catalyst for strand invasion, homology search, and pairing of DNA during HR. Both BARD1 and BRCA1 are capable of physically interacting with RAD51 and DNA; however, it is the BRCA2-DSS1 complex that facilitates the replacement of RPA with RAD51 [49]. PALB2 binds both BRCA1 and BRCA2 and functions as a bridge between them. BRCA1 and PALB2 interact via their respective coiled-coil domains [50]. Once bound to BRCA1, the PALB2′s WD40 domain enables the recruitment of BRCA2-DSS1 to DSBs [51]. BARD1-BRCA1-PALB2 effectively serves as a scaffold for BRCA2-mediated RAD51 loading. Additionally, the binding of BARD1-BRCA1 to RAD51 is thought to aggregate RAD51 for improved efficiency and success of RPA replacement by BRCA2-DSS1, but this idea has yet to be tested [52]. The formation of the BARD1-BRCA1-PALB2-BRCA2-DSS1 complex occurs in a stepwise fashion. Mutations therein alter DNA foci in a parallel stepwise manner with BRCA1 mutations impairing the complex from forming entirely [53]. Thus, BARD1-BRCA1 is central to the recruitment of BRCA2-DSS1 to DSBs in HR.

### 2.3. BARD1-BRCA1 in Mismatch Repair

Both BARD1 and BRCA1 have been shown to interact with MSH2 and MLH2, two important mismatch repair (MMR) proteins [54,55,56]. These molecular interactions suggest that BARD1-BRCA1 may contribute to MMR. However, evidence supporting this is conflicted, as genetic studies suggest a functional interaction between BRCA1 and MSH2 in HR [56]. Overall, BARD1-BRCA1 involvement in MMR is underexplored, but this connection could explain the incidence of colorectal cancer (CRC) in BRCA1- and BARD1-mutated CRC patients as MMR defects have long been known to predispose individuals to CRC [57].

## 3. BRCA1-Independent Functions of BARD1

### 3.1. Regulation of p53 and Apoptosis

BARD1 has been implicated in the regulation of apoptosis. BARD1 overexpression induces apoptosis, while the tumor-related mutation, Q564H, diminishes BARD1’s pro-apoptotic ability when exposed to genotoxic stress [58].

The function of BARD1-related apoptosis is associated with binding to p53, Ku70, and the phosphorylation of p53 at Serine-15 [15,58]. Binding of p53 occurs in the ANK-BRCT linker region and the BRCT domain [15,59,60]. In cell lines that are apoptosis-resistant and deficient in phosphorylated Serine-15 of p53 (NuTu-19 and HEK 293T cell lines), BARD1 overexpression can restore the phosphorylation capacity. This suggests that BARD1 promotes the formation of p53 and DNA-PK complexes, which then allows for p53 phosphorylation by ATM and results in apoptosis [15]. Additionally, mutations associated with breast, ovarian, and uterine malignancies lack sequences in the ANK-BRCT linker region that are vital for BARD1-dependent apoptosis [59]. BARD1 has also been shown to stabilize p53 in a slightly different setting. In cervical cancer progression, human papillomavirus (HPV) 16 E6 transforming protein aims to inactivate p53, however, BARD1 adds a layer of protection by increasing and stabilizing p53 while inactivating E6 [61].

BARD1 exerts its pro-apoptotic function in the cytoplasm after BARD1 is shuttled out of the nucleus [7,59]. Schüchner et al. discovered three NLS within BARD1 that are centrally located and do not overlap with other functional domains [8]. Just one NLS sequence is sufficient for translocating BARD1 into the nucleus, which is independent of BRCA1 [59]. BARD1 also contains a CRM1-dependent NES at the N-terminus following the RING domain, allowing for nuclear export through CRM1 [7]. Interestingly, BRCA1 suppresses BARD1 apoptotic activity by interfering with its export, shifting BARD1 towards its BRCA1-dependent cell survival activity [7,58]. Without the influences of BRCA1, BARD1 can localize to the cytoplasm and induce apoptosis [7,59].

Furthermore, Tembe et al. showed that BARD1-p53 localizes to the mitochondria where BARD1 disrupts the mitochondrial membrane potential and induces apoptosis [60,62]. BARD1 also has the ability to relocate to the mitochondria without p53, however, p53 likely acts as an intermediary in BARD1-related downregulation of Bcl-2 leading to Bax oligomerization [63]. BARD1’s ability to induce Bax oligomerization and apoptosis relies on an intact BRCT domain [60]. The deletion of its BRCT domain not only limits the ability of BARD1 to bind p53, but it also inhibits its export out of the nucleus and localization to the cytoplasm and mitochondria [60].

On the other hand, BARD1 promotes the import of BRCA1 to the nucleus, whereas p53 regulates BRCA1 export by interfering with BRCA1-BARD1 binding [63,64]. BRCA1, similar to BARD1, also induces apoptosis in the cytoplasm [7,60,64]. However, BARD1 inhibits the export of BRCA1 out of the nucleus and disrupts the ubiquitin E3 ligase activity, both of which disrupt BRCA1-dependent apoptosis [64]. Mutating the NLS of BARD1 results in BRCA1 localizing to the cytoplasm, thus supporting the hypothesis that BARD1 acts as a chaperone to transport BRCA1 into the nucleus [8].

### 3.2. Cell Cycle Regulation/Mitosis

The amount of BRCA1 protein increases during S-phase, while the level of BARD1 protein remains constant from G1 to G2 but increases during mitosis [65,66]. BARD1 does play a vital role in S-phase progression [66]. Cell cycle-dependent kinase complexes, CDK2-cyclin A1/E1 and CDK1-cyclin B, phosphorylate BARD1 resulting in potentiation of its function in mitosis [67].

Localization and overexpression of BARD1 in the nucleus, but not the cytoplasm, results in G1 cell cycle arrest [8]. Similar to the BARD1 location-dependent apoptosis described above, cell cycle regulation may be dependent on whether BARD1 is localized to the nucleus or the cytoplasm.

### 3.3. NF-κB

BARD1 can also bind *NF-κB* and regulate its transcriptional activity [68,69]. BARD1, in addition to Pirin and Tip60, forms a Bcl-3 interacting proteins (BIP) network that then forms a quaternary complex with Bcl-3 and p50 [68]. Specifically, from half of the ANK into the BRCT domains, BARD1 binds the ANK repeats of Bcl-3 [68]. The complex then interacts with the promoter of the *NF-κB* gene and activates its transcription. Regarding BRCA1, BARD1 has been shown to inhibit the ability of BRCA1 to activate transcription through NF-κB [70].

### 3.4. Inhibition of mRNA Processing in Response to DNA Damage

A polyadenylation factor, CstF-50, has been shown to bind BARD1 in the nucleus to inhibit polyadenylation of mRNA [71]. CstF-50 binds the ANK-BRCT linker region of BARD1, thus involving BARD1 in mRNA processing and stabilization of RNA polymerase II (RNAP II) in response to DNA damage [6,71,72]. Within the RNAP II holoenzyme, BARD1 can recognize sites of DNA damage, and, through inhibition of polyadenylation of mRNA, BARD1 acts to prevent the processing of immature transcripts that may otherwise be translated to deleterious proteins [71,73]. The inhibition of transcription is further ensured through BRCA1-BARD1 mediated ubiquitination of RNAP II [74]. In response to DNA damage, cells have decreased levels of polyadenylation with concurrent increases in the CstF-50-BARD1-BRCA1 complex [73]. BARD1 is phosphorylated by CDK-cyclin at Threonine-734, which is important for its interaction with CstF-50 [65,75]. The role of BARD1 in mRNA processing has implications in tumorigenesis. For example, the Q564H mutation of BARD1 found in ovarian, breast, and uterine tumors, reduces the BARD1-CstF-50 interaction, and prevents their inhibition of polyadenylation [73]. Interestingly, p53 associates with BARD1-CstF-50, and tumor-related mutations in p53 also result in decreased BARD1-CstF-50 association and inhibition of mRNA cleavage [76].

### 3.5. ADP Ribosylation, Poly (ADP-Ribose) Polymerase and BARD1

The transfer of ADP-ribose from NAD^+^ to a target protein is called ADP ribosylation. A group of enzymes, called poly (ADP-ribose) polymerases (PARPs), further catalyze the polymerization of ADP-ribose (PAR) [77]. These later reactions are termed PARsylation. Nuclear PARPs play a critical role in DDR and genome stability [78]. Inhibition of PARPs has been implemented in the treatment of *BRCA1/2* mutated cancers [79,80,81,82,83]. BARD1 plays an essential role in the PAR signaling in response to DNA damage. Specifically, BARD1’s BRCT domain directly binds the ADP-ribose within PAR and recruits BRCA1 to the damage sites [79].

## 4. BARD1 in Non-Breast and Non-Gynecological Cancers

### 4.1. Neuroblastoma

Neuroblastoma (NB) is a tumor that arises from the sympathetic nervous system and accounts for approximately 10% of pediatric cancers and 15% of childhood deaths related to cancer [84]. Genome-wide association studies (GWAS) in NB have shown that BARD1 acts as a tumor suppressor during its development and that certain variations in a single nucleotide have profound effects on BARD1 protein expression and NB susceptibility (Table 1). Initially, Capasso et al. identified multiple SNPs in the *BARD1* gene from blood samples of NB patients from European American and European populations [85,86]. One particular SNP, rs6435862 T > G, which is located in intron 1 of the *BARD1* gene and results in splicing of exon 2 and 3 and formation of BARD1β, was most significantly associated with susceptibility to NB. Their data was later replicated in African American, Italian, and Han Chinese populations [86,87,88,89]. Studies also found that rs6435862 was associated with high-risk NB [85,86,87], suggesting that BARD1β is an oncogene [90]. In European American blood samples, this variant was present in stage IV, MYCN amplified NB and associated with diagnosis after age 1.5 years [86]. The Italian cohort showed a similar trend in terms of age at diagnosis, high-risk classification, and MYCN status, but did not reach statistical significance. In the Han Chinese population, rs6435862 was associated with stage IV tumors and adrenal gland as the primary site while another study in Han Chinese showed additional associations with stage III disease and the onset of NB after one year of age in patients homozygous for the G risk allele [88,89]. Collectively, these data strongly suggest that rs6435862, which results in the expression of the oncogenic BARD1β isoform, is associated with poor prognosis of NB.

Knockdown of BARD1β in NB cell lines, NLF and Nb-Ebc1, which contain rs6435862, resulted in significant inhibition of proliferation and colony formation [90]. The enhanced proliferation and evasion of apoptosis in NB caused by this variant were through BARD1β interaction with and stabilization of Aurora kinases A and B. The mechanisms of BARD1β were independent of p53 and BRCA1-dependent HR because silencing of BARD1β did not alter the level of phosphorylated p53 and additional silencing of PARP1 was not lethal to NB cells. Intriguingly, selective serotonin reuptake inhibitors (SSRIs), citalopram and escitalopram, inhibited survival and induced apoptosis of NB cell lines regardless of MYCN status [91]. It was proposed that the decrease in viability was caused by inhibition of BARD1β, which normally stabilizes Aurora kinase A, which then stabilizes MYCN, a major oncogenic driver in NB. These studies suggest that NBs due to the expression of oncogenic BARD1β can potentially be treated with Aurora kinase inhibitors.

Another intronic SNP associated with the development of NB is rs3768716. Combined tumor samples from the United Kingdom and the United States found that this variant was associated with high-risk NB with an odds ratio of 1.68 [85]. A Chinese population with this variant also showed a significant risk of developing NB [88], while a second Chinese study and an African American cohort exhibited increased tendency but did not reach significance [87,89]. rs3768716 could indicate an aggressive disease phenotype since the SNP was associated with stage III and IV NB, origination at the adrenal gland and diagnosis after 12 months of age [88,89].

One of the most common SNPs in the *BARD1* gene associated with NB is rs17489363, which is located in the promoter region [88,92]. This variant decreases the transcription of BARD1-FL. One of the rs17489363 variants, which converts C to T risk allele, was associated with high-risk NB among European Americans, African Americans, Italians, and Spaniards [92,93]. High-risk NB had significantly decreased mRNA expression of BARD1-FL compared to intermediate- and low-risk NB [92]. Knocking down of BARD1-FL in SHSY5Y and SKNSH, two human NB cell lines, led to increased viability and invasion, which is consistent with the tumor suppression function of BARD1-FL in NB. Heat shock factor 1 (HSF1) bound more strongly to the *BARD1* gene with homozygous T risk allele of rs17489363 present in the SHSY5Y cell line compared to its homozygous C allele in the SKNAS cell line. rs6720708, another SNP in the *BARD1* gene, has the strongest association with NB arising from the adrenal gland [94]. Since it is in strong linkage disequilibrium with rs17489363, it is likely that rs17489363 is also associated with this site of origin. The second rs17489363 variant, which converts G to A risk allele, was found in an NB susceptible Chinese population [88]. The A risk allele was associated with origination from the adrenal gland. Furthermore, clones containing the A risk allele decreased transcription of BARD1-FL by 1.2- to 4-fold compared to those containing the G allele. Taken together, the A and T risk alleles demonstrate similar phenotypes. rs17489363 predisposes to NB originating from the adrenal gland by allowing the binding of HSF1 to the *BARD1* gene at the promoter region and reducing the transcription of BARD1-FL, thereby affecting its DDR function.

On the other hand, other SNPs in the *BARD1* gene have been shown to be negatively associated with NB susceptibility and therefore may play a protective role. rs7585356, located downstream of the *BARD1* gene in the 3′ untranslated region (UTR), resulted in overexpression of BARD1-FL [85,86,89]. This variant was negatively associated with high-risk, stage IV NB as well as MYCN amplification, and more commonly diagnosed at an age of less than 18 months in European Americans [86]. DNA taken from Italian children with NB showed a similar association with disease stage and age of diagnosis. Harboring at least one A allele at rs7585356 was significantly associated with decreased risk of NB in Chinese girls [89]. rs7585356 by conversion of the G allele to an A allele overexpressed BARD1-FL and resulted in better clinical prognosis. rs1048108, located in exon 1 of the *BARD1* gene near the RING domain, was also negatively associated with NB risk in European Americans, African Americans, Italians, and Chinese people [85,88,92]. Pathway analysis indicated that rs1048108 repressed cellular development and regulated apoptosis [95]. This variant showed no differences in its binding to BRCA1 compared to the wild-type counterpart, indicating that the protective role of the BARD1 SNP, rs1048108, was independent of BRCA1 [88].

Ethnic disparities exist amongst NB prevalence and severity. In the United States, NB is more common in children of European descent; however, the high-risk disease is more prevalent in children of African descent [87]. Latorre et al. identified BARD1 SNPs for the first time in African American patients that were not reported in European Americans. For example, rs16852804 was significantly associated with NB susceptibility, while rs7599060 was significantly associated with high-risk disease. Studies in a Chinese population identified a variant, rs3738888, that was unique to NB susceptibility in Han ethnicity [88]. This indicates that ethnicity may play an important role in genetic susceptibility to NB. There are many other single nucleotide variants of BARD1 that are significantly associated with NB susceptibility (Supplemental Table S1), however, the details regarding their role in cancer prognosis still need to be further investigated.

In summary, BARD1-FL acts as a tumor suppressor in the development of NB, primarily by regulating DDR, cellular proliferation, and programmed cell death. BARD1-FL promotes DDR through G2-M checkpoint arrest via downregulation of cyclin B and induction of apoptosis via phosphorylation of p53 [96]. It is hypothesized that the cell cycle arrest is through the BARD1-BRCA1 heterodimer while cell death was BRCA1-independent, but the mechanisms were not directly tested. SNPs in the *BARD1* gene have both oncogenic and tumor-suppressing roles. In support of BARD1 as a tumor suppressor, rs7585356 leads to the overexpression of BARD1-FL while rs1048108 does not affect BRCA1 binding. Both SNPs are associated with a decreased risk of NB. Reduced expression of BARD1-FL (rs17489363) and formation of the oncogenic BARD1β isoform (rs6435862) increase susceptibility to NB and correlate with poor prognostic factors such as stage III and IV disease, older age at diagnosis and tumor originating in the adrenal gland. BARD1β affects DDR in a BRCA1-independent mechanism. The proliferation of NB cell lines and xenograft tumors with mutations or deletions in DNA repair genes, including BARD1, were inhibited by the PARP inhibitor (PARPi), Olaparib [97]. Thus, SNPs leading to oncogenic isoforms or decreased activity of BARD1-FL result in dysregulation of DDR, and those patients could benefit from treatment with Aurora kinase inhibitors or PARP inhibitors.

### 4.2. Gastrointestinal Cancers

#### 4.2.1. Normal Colon and Colorectal Cancers

Colorectal cancer (CRC) is the third leading cause of cancer-related deaths in the United States [98] and worldwide [99]. Dysfunction of DNA repair and cell cycle regulators have been implicated as the oncogenic drivers for the development of CRC [100]. For example, mutations in the MutS homolog 2 (MSH2), a mismatch repair protein, are prevalent in Hereditary Non-Polyposis Colorectal Cancer as well as sporadic cases. Wang et al. showed that BRCA1 and BARD1 interact with MSH2 in a region that overlaps with its adenine nucleotide-binding (ANB) domain, to which two other mismatch proteins, MSH3 and MSH6, bind [54]. BARD1 also interacts with MSH3 and MSH6 suggesting that BARD1-BRCA1 directly interacts with both MSH2-MSH3 and MSH2-MSH6. These interactions were further demonstrated by the colocalization of MSH2-MSH6 and BRCA1 at DNA damage sites after cisplatin treatment. The ANB domain of MSH2, which exchanges ADP for ATP when MSH2-MSH6 or MSH2-MSH3 identify and bind the mismatched nucleotides, is necessary for the recruitment of BRCA1 and thus BARD1 for DNA repair. Therefore, mutations in BARD1 may play a role in CRC tumorigenesis through the disruption of MMR.

Many variants of BARD1 have been identified and characterized in CRC. Sporn et al. identified 19 splice variants, which removed exons, from 15 colon cancer samples with matched normal colon tissues [101]. Comparison of these matched tissues showed that the variants which removed exon 4 (4a/5, 3/5, 1/5) were decreased in tumor compared to the normal colon; however, in general, variants 4a/5 and 3/5 are more highly expressed in normal tissue. Thus, the pathogenicity of these variants still needs further investigation. A second study using 10 matched paired samples found that BARD1-FL and all its isoforms were significantly upregulated in tumors compared to normal colonic tissue [14]. Isoforms ϕ, δ, and π were associated with age greater than 60 years old while κ associated with tumor growth to at least the outer lining of the bowel, stage III and IV disease and lymph node metastasis. Immunohistochemistry (IHC) staining of 168 tumors was performed using antibodies to detect regions corresponding to exons 1, 3, 4, and 11. The most common staining pattern was the detection of antigens designated to exons 3 and 4 simultaneously, which is indicative of the π isoform, and correlated with decreased survival. Positive staining for antigens mapped to either exon 3 or exon 4 showed no improvement in survival, suggesting that isoforms β, κ, and π may promote tumor growth in CRC.

Although CRCs expressing BARD1β showed poor prognosis, a new treatment is promising for patients expressing this isoform. CaCo-2 cells, which express BARD1β and wild-type BRCA1, showed sensitivity to PARPi with an IC_50_ of ~17.5 uM [102]. PARPi treatment of SW480 cells transfected with BARD1β increased the number of γH2AX foci, decreased the number of RAD51 foci, and reduced the percent of cells in the G1 phase of the cell cycle. As this trend is similar to the CaCo-2 cells, BARD1β causes PARPi sensitization likely through disrupting HR. The pathogenicity of BARD1β in CRC is mediated through BRCA1. The amount of BRCA1 present in the nucleus was much higher in the control SW480 cells compared to their BARD1β-expressing counterparts. Cellular ubiquitin ligase activity was drastically reduced in the BARD1β-expressing cells as indicated by decreased ubiquitination of MYC after treatment with proteasome inhibitor MG132. Furthermore, the phenotype of the BARD1β-expressing cells showed more metastatic potential. BARD1β-expressing cells had a greater ability to migrate and form colonies, which was supported by their epithelial-mesenchymal transition (EMT) expression profile: increased β-catenin and decreased E-cadherin, snail, and vimentin. Nonetheless, PARPi therapy predicts a favorable outcome for patients expressing the aggressive BARD1β isoform.

BARD1 9′L is a unique alteration of the *BARD1* gene, in which transcription begins in intron 9 and the mRNA includes parts of exon 10 and 11 and encodes a long non-coding RNA (lncRNA) [103]. BARD1 9′L binds to the 3′ UTR of *BARD1* and counteracts the actions of microRNA (miR)-203 and -101 at this site, which normally reduce the mRNA expression of BARD1-FL and the β, δ, and γ isoforms. The possible pro-tumorigenic role of BARD1 9′L was supported by increased expression in colon tumors in comparison to matched non-cancerous tissue.

Given that a multitude of variants in BARD1 have been identified in CRCs and normal colons, both tumorigenic and protective alterations exist. In general, expression of BARD1-FL, certain isoforms, and the C-terminal regions seem to inhibit carcinogenesis. IHC staining for BARD1-FL in 81 CRC tissues showed that lower expression of BARD1-FL correlates with stage IV disease and poor survival while higher expression correlates with better survival [101]. However, Zhang et al. argued that the antibody used in previous studies was against the first 300 a.a. of BARD1 and could also detect the π, γ, δ, ϕ, and ε isoforms [14]. In their study, positive staining with four antibodies detecting different regions of the BARD1 protein, only possible with the expression of BARD1-FL, increased survival. However, lack of BARD1 promoter methylation indicated that oncogenic isoforms are responsible for tumorigenesis instead of downregulation of BARD1-FL. Positive staining of tumors for antigens mapped to either exon 1 or 11 was significantly associated with increased survival and their expressions were correlated. Staining patterns with co-expression of only these N- and C-terminal regions is consistent with the δ and ϕ isoforms, suggesting that these variants may prevent tumor growth. Additionally, the silencing of BARD1γ, which is comprised of exons 1–3 and contains the RING domain, decreased the expression of BARD1-FL, suggesting that BARD1γ may help to stabilize BARD1-FL.

Finally, apoptotic bodies, derived from tumor cells treated with sodium butyrate and IL-2 combination, cured rats of peritoneal colon carcinomatosis [104]. The apoptotic bodies contained a 67 kDa fragment of BARD1 containing the ANK repeats and the BRCT domain but lacking the RING domain. Sodium butyrate treatment of SW48 cells showed that BARD1 cleavage occurs early in apoptosis during the G0/G1 phase and is mediated by calpain. The importance of the ANK repeats and the BRCT domain are further supported by pathogenic variants disrupting this region. An extremely rare variant of *BARD1* with strong CRC inheritance pattern, c.1811-2A > G, resulted in the removal of exon 9 due to exon skipping, which is part of the BRCT domain [105]. Missense mutations, *BARD1* c.1217G > A p.Arg406Gln (rs587780014) and *BARD1* c.1918C > A p.Leu640Ile (rs1553612535), were present in three patients with stage III or IV CRC diagnosed before the age of 50 [106]. As these mutations are located in or near the ANK and BRCT domains, etoposide treatment of patients’ lymphoblastoid cells did not hinder BARD1 and BRCA1 colocalization or RAD51 foci formation. However, the mutants had fewer apoptotic cells after treatment with etoposide compared to the healthy controls. Thus, these variants may inhibit apoptosis through disruption of the interaction between p53 and the ANK repeats and BRCT domain. 

In conclusion, CRC initiation is largely attributed to the inability to repair DNA damage, which can be caused by BARD1 variants that prevent BRCA1 interaction at its RING domain, such as with BARD1β. However, mutations in the ANK and BRCT domains have been shown to disrupt other protein-protein interactions, possibly to p53. Therefore, the pathogenic variants of BARD1 in CRCs could be both BRCA1-dependent and BRCA1-independent. 

#### 4.2.2. Esophageal Squamous Cell Carcinoma

Esophageal squamous cell carcinoma (ESCC) has a 5-year survival rate of approximately 10% [107]. The current standard of care includes surgical resection, chemotherapy, and radiation therapy. Resistance to DNA damaging related therapies has been attributed to the upregulation of BARD1 [108,109]. Cisplatin resistance of Eca109 and TE-1, two ESCC cell lines, was due to the upregulation of integrin α5, which activated the PI3K/AKT pathway via phosphorylation of FAK and SRC resulting in survival [109]. In turn, PI3K upregulated the expression of BARD1, which then enhanced DDR as measured by decreased γH2AX foci and conferred chemoresistance. Additionally, knocking down of Neutrophin Receptor-Interacting MAGE Homolog (NRAGE) led to susceptibility to cisplatin, etoposide, and irradiation [108]. The silencing of NRAGE caused the ubiquitination of RING Finger Protein 8 (RNF8) and BARD1. NRAGE formed a ternary complex with RNF8 and BARD1 and inhibited their degradation. The RING domain of BARD1 interacts with NRAGE while its BRCT domain interacts with RNF8. NRAGE and RNF8 also interact with each other. Upregulation of the NRAGE-BARD1-RNF8 complex increased HR and caused resistance to radiation and chemotherapies, which kill cancer cells by inducing DSBs.

Given that the mechanism of action of most chemotherapy and radiation therapy is the induction of DNA damage, it is not surprising that DDR genes were upregulated in the resistant cells. The cisplatin-resistant cells showed upregulation of BARD1 and BRCA1, and knocking down of integrin α5 resulted in a significant decrease in mRNA expression of both genes [109]. Similarly, the silencing of NRAGE showed increased γH2AX foci, which colocalized with those of BRCA1 and 53BP1 [108]. Although NRAGE-expressing ESCC cells did not have increased BRCA1 protein expression compared to NRAGE-silenced cells, RNF8 regulated BRCA1 recruitment while BARD1 formed a heterodimer with BRCA1 to increase HR. This suggests that resistance to chemotherapy and radiation therapy in ESCC is at least partially mediated by enhanced HR likely through the BARD1-BRCA1 complex, however, more studies need to be conducted to confirm whether BARD1 triggered DDR and survival through BRCA1-dependent or -independent mechanisms, or both.

#### 4.2.3. Hepatocellular Carcinoma

85% of hepatocellular carcinoma (HCC) cases are preceded by cirrhosis [110], and the high worldwide incidence is largely attributed to viral infection (hepatitis B and C, or HBV and HCV) or fatty liver (alcoholic and non-alcoholic) [98,110]. Therefore, detecting early changes is important as surveillance for the development of liver cancer in cirrhotic patients. Lubecka et al. examined DNA methylation in white blood cells (WBCs) of HBV-negative populations and detected pre-diagnostic epigenetic changes that could be utilized for identification of those at risk for the development of HCC [110]. Patients with pre-diagnosed HCC had significant hypomethylation of the *BARD1* gene compared to healthy patients as measured by a 13.3% difference between the groups. A similar difference was identified in cirrhotic patients who eventually developed HCC compared to those who never developed cancer. Gene expression studies showed significant overexpression of BARD1 in the HCC cohort compared to cirrhotic controls and a similar trend was seen with healthy controls. Later in the disease (after diagnosis), the *BARD1* gene was methylated and showed similar mRNA expression to controls. They thus proposed that the *BARD1* gene hypomethylation, among epigenetic changes of 8 other genes, can be used as an identifier for the likelihood of HCC with predisposing risk in HBV-negative patients.

Liao et al. found significant differences in BARD1 mRNA and protein expression in HCC tumor samples compared to adjacent cancer-free tissues [111]. The majority of tumors highly co-expressed BARD1 and alpha-fetoprotein (AFP), which improved diagnostic sensitivity to ~84%. BARD1 expression was significantly associated with poor prognostic factors such as TNM stage III and IV, Barcelona clinic liver cancer (BCLC) stage B and C, tumor size greater than 5cm, HBV infection (positive HBV surface antigen, or HBsAg) and high serum AFP and aspartate aminotransferase (AST) concentrations. HCC patients with higher expression of BARD1 had significantly decreased progression-free survival (PFS) and overall survival (OS) (PFS ~35 months; OS ~40 months) compared to lower BARD1 expression in their tumors (PFS ~53 months; OS ~57 months). Mechanistically, the knocking down of BARD1 in SMMC7721 and Huh7, two HCC cell lines, decreased colony formation, invasion, and migration. BARD1 silencing also downregulated the level of total AKT and phospho-AKT as well as its downstream effectors, such as total mTOR, phospho-mTOR, and MMP9, suggesting that HCC tumor survival is mediated through BARD1 activation of the AKT pathway and is independent of p53. Since BARD1-FL acts as a tumor suppressor, these studies suggest that the BARD1 they are detecting is likely mutated or a splicing variant, however, more research needs to be conducted to classify the isoforms of BARD1 that play an oncogenic role in HCC.

#### 4.2.4. Pancreatic Cancer

Pancreatic cancers are the fourth leading cause of death due to cancer in the United States and have a 5-year survival of approximately 9% [98]. Pancreatic ductal adenocarcinomas (PDAC), which comprise the vast majority of pancreatic tumors, arise from the exocrine cells of the ducts and are typically associated with heritable syndromes such as hereditary breast and ovarian cancer syndrome and Lynch syndrome [112]. Sequencing of lymphocyte DNA from 302 PDAC patients with a positive family history of PDAC showed that 11.9% of the patients had pathogenic variants, of which 25% were not known to be associated with PDAC [113]. A novel mutation in the *BARD1* gene was discovered in a patient who had 5 family members with PDAC but no other cancer types. *BARD1* c.632T > A (p.Leu211*) results in a premature stop codon. In a similar study, 96 patients with PDAC, regardless of familial status, were sequenced, and 13.5% of the patients had germline pathogenic SNPs [114]. They identified a mutation in *BARD1* c.1921C > T p.Arg641X in a PDAC patient who had one relative with pancreatic cancer that resulted in a premature stop. Interestingly, this SNP (rs587781948) was also found to have an increased risk of NB (Supplemental Table S1) [115,116]. Thus, these truncating mutations that result in the inactivation of BARD1, likely increase susceptibility to heritable PDAC. Additionally, analysis of lymphoblasts from 100 familial pancreatic cancer patients detected two SNPs in BARD1, rs2229571 and rs1129804, which had profound effects on its gene expression (greater than 4-fold change) [117]. Although the function of the variants was not investigated, these SNPs are likely associated with susceptibility to familial pancreatic cancer since rs2229571 increased the risk of NB (Supplemental Table S1) [85,86,88].

Neuroendocrine neoplasms (NEN) originate from neuroendocrine cells arising from various tissues, the most common of which is the gastro-entero-pancreatic site [118]. Most NENs are classified based on germline mutations resulting in hereditary syndromes or from IHC staining patterns. The remaining tumors are unclassified and rely on genetic sequencing to identify the oncogenic driver and therapeutic target. Genetic testing has identified mutations in many genes involved in the BRCA-associated HR pathway [119]. One patient with an unremarkable family history for cancer who developed a pancreaticoduodenal tumor with metastasis to the liver and abdominal lymph nodes was found to have a germline alteration in the *BARD1* gene: c.69_70delins25 (p.Ala25Glyfs*41) [119]. This deletion-insertion is thought to cause a truncated form of BARD1 due to a premature stop codon. Thus, mutations in *BARD1* can lead to the development of pancreatic cancers originating from neuroendocrine cells.

Novel germline mutations in the *BARD1* gene have been identified in pancreatic cancers, arising from either neuroendocrine or exocrine ductal cells, however, their function and role in susceptibility have yet to be validated. Zhang et al. applied a computational approach utilizing pancreatic cancer-associated genes and pathway profiles of 79 pancreatic tumor samples and discovered *BARD1* as one of six candidate genes for the development of pancreatic cancer [120]. BARD1 was related to the other six candidate genes through its interaction with BRCA2. Pancreatic cancers that highly expressed these six genes demonstrated worse overall survival compared to those patients with lower expression. Although the susceptibility to PDAC and pancreatic NEN in patients with rare germline mutations in BARD1 remains to be studied, familial inheritance patterns and research evidence suggest that BARD1 may disrupt HR as mutations in BRCA2 and other DDR genes have also been shown to be tumorigenic.

### 4.3. Non-Small Cell Lung Cancer

Lung cancer is the primary cause of cancer-related death worldwide [121]. Lung cancer is subdivided into two main categories: small cell lung cancer (SCLC) and non-small cell lung cancer (NSCLC). NSCLC is comprised of adenocarcinoma, squamous cell carcinoma, and large cell carcinoma. Zhang et al. identified nearly all isoforms (β, γ, δ, ϕ, ε, η, κ, π) of BARD1 from RNA extracted from 20 matched NSCLC tumor and normal adjacent tissue samples [18]. BARD1-FL and all other detected isoforms were increased in tumors compared to neighboring normal lung tissue with the π isoform being the most significantly upregulated in tumors. BARD1 was undetected in normal healthy controls, suggesting that BARD1 and its isoforms play a key role in cellular transformation and tumor progression in the lung. Stratification by sex indicated that β and κ isoforms were expressed higher in males while the η, γ, and ε isoforms were higher in females but did not reach statistical significance. Additionally, the lncRNA BARD1 9′L, which stimulates the expression of BARD1-FL and isoforms β, δ, and γ through interaction with the 3′ UTR of *BARD1*, was significantly upregulated in lung tumors compared to paired cancer-free lung tissue as well as in the human lung adenocarcinoma cell line, A549 [103].

IHC staining analyses of 54 NSCLC tumors using antibodies that detect antigens mapped to exon 1 and exon 11 of BARD1 showed the highest expression of BARD1 in large cell carcinoma followed by squamous cell carcinoma then adenocarcinoma [122]. Staining patterns varied and rarely showed positivity for all antibodies used, indicating the presence of different isoforms in NSCLC [18,122]. An additional 100 NSCLC tumors were stained with antibodies recognizing antigens mapped to exons 1, 3, 4, and 11 [18]. Staining for regions corresponding to exons 1 and 11 correlated and indicated the presence of ϕ, δ, and ε isoforms. The π isoform was also detected as staining for regions mapped to exons 3 and 4 also correlated. Expression of either exon 3, or beginning of exon 4, or both, present in isoforms β, κ, and π, was associated with decreased disease-free survival and overall survival. These isoforms were also the most prevalent and more common in squamous cell and large cell carcinomas compared to adenocarcinoma. When BARD1β was overexpressed in A549 cells, it increased cellular proliferation, inhibited apoptosis, and increased the expression of fibronectin, an inducer of EMT, compared to its BARD1-FL expressing counterparts [123]. Intriguingly, the expression of BARD1 isoforms did not correlate with tumor grade or stage [18,122]. However, induction of lung adenocarcinomas in mice led to the detection of staining patterns positive for antigens corresponding to exon 1 in early disease and exons 3 and 4 in the later disease that were higher in tumors compared to adjacent tissues [18]. Thus, the π isoform is likely involved in tumor progression and an aggressive phenotype.

Since the BARD1 isoforms are highly expressed in NSCLCs, Pilyguin et al. proposed that the detection of antibodies in patients’ sera against the tumor-associated antigens present on the BARD1 isoforms could be used to predict the presence of lung cancer [124]. They were able to distinguish between those with lung cancer and healthy individuals with high sensitivity and specificity with a model using 27 antigens and patients’ sera. The same 27 antigens model did not predict NB, ovarian, or breast cancer based on a sensitivity of at least 0.90, although some samples were detected. In addition, applying the ten highest expressed peptides to stages I-III or stage IV disease showed no differences. Therefore, the simple detection of antibodies against BARD1 isoforms in blood sera can accurately predict the presence of lung cancer, regardless of stage.

One proposed treatment modality for patients with NSCLC is the combination of neddylation and PARP inhibitors [125]. The neddylation E1 inhibitor, MLN4924, decreased BARD1 intensity following laser ablation. The combination treatment of MLN4924 and Olaparib for 72 h significantly inhibited the proliferation of A549 and H1299 NSCLC cells. Irradiation following the combination treatment resulted in defective DNA repair, as indicated by a significantly increased number of cells with γH2AX foci. Cell death was induced in NSCLCs by a combination of PARP and neddylation inhibitors, which caused and prevented repair of DSBs, respectively.

IHC staining of NSCLC tumors showed that the expression of BARD1 did not correlate with that of BRCA1 or p53 [18,122], suggesting that disruption of BARD1’s protein-protein interactions plays a role in tumorigenesis. Aurora kinase B was also upregulated in lung tumors [18]. Although the molecular pathways activated or inhibited by the BARD1 oncogenic isoforms in NSCLC need to be further investigated, inhibition of these mutated proteins, especially isoforms β, κ, and π, may drastically inhibit tumor progression.

### 4.4. Other Cancer Types

#### 4.4.1. Nephroblastoma

Nephroblastoma, also known as Wilms’ tumor, is a pediatric tumor of embryonal origin that arises in the kidney and accounts for 6–7% of cancers in children [126]. Although the prevalence of nephroblastoma is lowest in Asian ethnic groups, Fu et al. examined whether three variants in the *BARD1* gene were associated with 145 nephroblastoma patients compared to 531 cancer-free controls from an ethnic Han population from Southern China [127]. rs7585356 homozygous for the A risk allele significantly increased the risk of nephroblastoma, specifically stage I and II disease, when compared to the wildtype G alleles. While there was a trend toward increased risk with an increased number of variants, patients with three risky variants in *BARD1* (rs7585356, rs6435862, and rs3768716) were significantly associated with nephroblastoma susceptibility with an odds ratio of 2.21, and subgroup analysis identified increased risk in females and development of stage I and II disease. This single study highlights the need for identification of functions of aberrant *BARD1* in nephroblastoma initiation and progression, particularly by expanding patient samples to include other ethnic groups and analyzing the frequency of more SNPs in the *BARD1* gene.

#### 4.4.2. Ewing Sarcoma

Ewing sarcoma, which is most common in Caucasian males in the second decade of life, is a pediatric cancer originating in bone, typically the pelvis and long bones [128]. The vast majority of Ewing sarcoma patients have chromosomal translocation *t*(11;22) producing the fusion protein EWS-FLI1 [128,129]. The C-terminus of Ewing sarcoma RNA binding protein 1 (EWS) binds to and regulates RNA processing [129]. The N-terminus of EWS activates transcription when bound to a DNA-binding domain-containing protein, such as the Friend Leukemia Integration 1 transcription factor (FLI1). Although EWS-FLI1 is the major oncogenic driver in Ewing sarcoma, transformation still occurs when the DNA-binding domain is lacking and is thought to be through protein-protein interactions of the N-terminus of EWS. The N-terminus of EWS and EWS-FLI1 interacts with the C-terminus of BARD1. Although it is hypothesized that BARD1-EWS recruits CstF-50 and alters mRNA processing, the function of this interaction still needs to be further elucidated. Recently, the first germline variation in the *BARD1* gene was identified in a patient with relapsed Ewing sarcoma [130]. A frameshift mutation in *BARD1* c.176_177AG; p.E59Afs*8 occurred in the RING domain. Interestingly, the patient’s father also harbored the *BARD1* germline mutation and had many relatives with early-onset breast cancer. This study suggests that the dysfunction of HR may be implicated in the familial inheritance of Ewing sarcoma. The interaction of the major oncogenic driver, EWS-FLI1, with the C-terminus of BARD1 as well as a frameshift deletion in the BRCA1-binding region of BARD1 in Ewing sarcoma strongly support the need for more functional studies of impaired DDR in Ewing sarcoma.

#### 4.4.3. Leukemia

The detection and characterization of oncogenic isoforms in BARD1 were recently reported in acute myeloid leukemia (AML), a cancer affecting the precursors of erythrocytes, granulocytes, monocytes, and platelets in adults [131]. Lepore et al. demonstrated significant upregulation of the protein fragment of BARD1 between exons 6 and 11, corresponding to the FL, α, β, κ, and π isoforms, in AML patient blasts and in AML cell lines, NB4, U937, K562, and HL60 [17]. Treatment with the histone deacetylase (HDAC) inhibitor, Vorinostat, decreased BARD1 expression suggesting that specific isoforms are upregulated in AML. Further characterization of three BARD1 truncated variants, which they called ω isoforms, in NB4 cells showed loss of the N-terminal region but retaining of the BRCT domain with or without the ANK repeats. Vorinostat induced apoptosis through increased expression of miR-19a and -19b, which directly bind to the 3′ UTR of *BARD1*, thereby decreasing its expression. Expression of the BARD1ω1 isoform that begins at the end of exon 4 at nucleotide 971 disrupted mitosis and directly interacted with p53 without inducing apoptosis. Knocking down of the BARD1 isoform drastically increased the number of apoptotic cells after Vorinostat treatment. Thus, future studies should examine the roles of other isoforms present in leukemia and the effectiveness of BARD1ω isoform inhibition to induce cell death in a p53-dependent manner.

#### 4.4.4. A Brief Summary of Genetic Changes in BARD1 Gene in a Variety of Cancers Identified through the Cancer Genome Atlas (TCGA) Project

According to the most recent TCGA database (as of July 2020), 169 *BARD1* mutations have been identified in a variety of cancers, including those discussed here. Of those mutations, the vast majority (150/169) were missense mutations. The mutations were distributed throughout the *BARD1* gene. Somewhat similar to what was observed in the literature, the majority of genetic mutations occurred in gynecological cancers, specifically uterine cancers, followed by cancers of the gastrointestinal tract, then lung cancers. Mutations in the *BARD1* gene were appreciated in 20–25% of tumor samples taken from the pancreas and the adrenal gland; however, the cohorts were very small and may not reflect the true prevalence of *BARD1* mutations in pancreatic cancer and NB. Of 125 reported mutations within the protein-coding region of *BARD1*, roughly 80% of the mutations were predicted to have moderate effects while the remaining 20% may have a relatively high impact on the biological functions of BARD1.

Utilizing the TCGA datasets, Adamovich et al. evaluated 76 possibly pathogenic *BARD1* mutations in 24 cancers [132]. The missense mutations were most prevalent in breast and uterine/endometrial cancers. BARD1 variants were also commonly found in cancers of the ovaries, prostate, brain, lung, and gastrointestinal tract. Pathogenicity was determined by at least a 40% reduction in DSB repair utilizing a functional HR assay. Of the 76 variants, 16 mutations had defective HR. Surprisingly, none of the variants in the RING domain led to functional loss of HR. Four mutations in the ANK region with defective HR were identified, suggesting that the ANK region plays an important role in DDR. More functional studies need to be conducted to determine the pathogenicity of different BARD1 variants in various cancer types.

## 5. Conclusions

In this review, the role of BARD1-FL as well as different SNPs in *BARD1* gene and alternatively spliced forms of BARD1 in non-breast and non-gynecological cancers were discussed and also summarized in Figure 2, Table 1, and Supplemental Tables S1 and S2. BARD1-FL has been widely accepted as a tumor suppressor [1,2,13,96]. In CRC and NSCLC, the expression of BARD1-FL was associated with increased survival [14,18,101], while the SNP rs7585356, which overexpressed BARD1-FL, was associated with decreased susceptibility to high-risk NB [86,89]. Nonetheless, the expression of BARD1-FL can have acquired oncogenic function in tumors where the upregulation of the DDR genes contributes to resistance to DNA damaging agents [108,109]. BARD1 isoforms caused by genetic mutations or SNPs are upregulated in many tumors [13,14,18,96,122]. Interestingly, few of the genetic mutations discussed here are present in and influence the risk of developing breast and gynecological cancers, suggesting that some of these *BARD1* SNPs may be cancer-specific (Supplementary Tables S1 and S2) [19,133,134,135,136,137,138,139,140,141,142,143,144,145,146,147,148,149,150]. Whether BARD1 variants are secondary to treatment or part of the oncogenic progression has not been fully elucidated. Therefore, developing inhibitors targeting the pro-tumorigenic BARD1 variants will be beneficial to sensitize those tumors to chemotherapy and radiation therapy, induce synthetic lethality with PARPi, or overcome secondary mutations that confer resistance to DNA damaging agents.

## Figures and Tables

**Table 1:** Common single nucleotide polymorphisms (SNPs) of the *BARD1* gene identified in neuroblastoma.

Neuroblastoma								
SNP	Susceptibility to NB	Location	Allelic Change	Function	Mechanism	Associates with	Population	Linkage Disequilibrium
rs6435862	↑	Intron 1	T > G	Removes exons 2 and 3 and produces the oncogenic BARD1β isoform [90]	BARD1β interacts with and stabilizes Aurora kinase A and B (independent of p53 and BRCA1) [90]	UK, AA/US: high-risk [85,87] EA/US: high-risk [85,86], stage 4N, MYCN amp, age > 18 mos. [86] Chinese: adrenal origin, stage IV, stage III & IV, age > 12 mos. [88,89]	European Americans [85,86], UK Caucasians [85], Italians [86], African Americans [87], Han Chinese [88,89]	
rs3768716	↑	Intronic	A > G			EA/US, UK, Italians: high-risk [85,86] Chinese: adrenal origin, stage III & IV [88,89], age > 12 mos. [89]	European Americans [85,86], UK Caucasians [85], Italians [86], African Americans [87], Han Chinese [88,89]	
rs17489363	↑	5′ UTR (promoter region)	C > T [92] G > A [88]	Decreases mRNA expression of BARD1-FL [88,92]	Through the binding of HSF1 [92]	EA/US, AA/US, Italians: high-risk [92] Chinese: adrenal origin [88]	European Americans, African Americans, Italians, Spaniards [92], Han Chinese [88]	rs6720708 [94]
rs6720708	↑		C > T			EA/US: adrenal origin [94]	European Americans [94]	rs17489363 [94]
rs7585356	↓	3′ UTR	G > A	Increases mRNA expression of BARD1-FL [86]		EA/US: high-risk, stage 4N, MYCN amp, age < 18 mos. [86] Italians: stage 4N, age < 18 mos. [86] Chinese: females [89]	European Americans [85,86], Italians [86], Han Chinese [89]	rs16852600 (intronic) [85,95]
rs1048108	↓	Exon 1 (P24S)	C>T [85,86] G>A [92]	Negatively regulates cellular development and modulates development and apoptosis [95]		EA/US, Italians, AA/US: high-risk [92]	European Americans [85,92], Italians [86,92], African Americans, Spaniards [92]	
rs16852804	↑	Intronic	C > T				African Americans [87]	
rs7599060	↑	Intronic	G > A			AA/US: high-risk [87]	African Americans [87]	
rs373888	↑	Exon 10 (R658C)	C > T			Chinese: adrenal origin [88]	Han Chinese [88]	

Arrows indicate increased (↑) and decreased (↓) susceptibility to NB.

## Supplementary Materials

The followings are available online at https://www.mdpi.com/2073-4425/11/7/829/s1, Supplemental Table S1: All reported SNPs of the *BARD1* gene detected in NB. Supplemental Table S2: All reported BARD1 variants in CRC, pancreatic cancer, NSCLC, nephroblastoma, Ewing sarcoma and AML.
