# Supplementary material for: The Effects of Genetic and Epigenetic Alterations of BARD1 on the Development of Non-Breast and Non-Gynecological Cancers
Source: Genes (Basel). 2020 Jul 21;11(7):829. doi: 10.3390/genes11070829 (PMC7396976; doi:10.3390/genes11070829)
Supplement: Supplementary file 1 [file genes-11-00829-s001.pdf]

Supplemental Table 1. All reported SNPs of *BARD1* gene detected in NB

**Neuroblastoma**									
SNP	Susceptibility to NB	Location	Allelic change	Function	Mechanism	Associates with	Population	Linkage dis-equilibrium	Presence in breast or gynecological cancers?
rs6435862	↑	Intron 1	T>G	Removes exons 2 and 3 and produces the oncogenic BARD1β isoform [90]	BARD1β interacts with and stabilizes Aurora kinase A and B (independent of p53 and PARP1) [90]	UK, AA/US: high-risk [85,87]<br>EA/US: high-risk [85,86], stage 4N, MYCN amp, age > 18 mos. [86]<br>Chinese: adrenal origin, stage IV, stage III & IV, age > 12 mos. [88,89]	European Americans [85,86], UK Caucasians [85], Italians [86], African Americans [87], Han Chinese [88,89]		
rs3768716	↑	Intronic	A>G			EA/US, UK, Italians: high-risk [85,86]<br>Chinese: adrenal origin, stage III & IV [88,89], age > 12 mos. [89]	European Americans [85,86], UK Caucasians [85], Italians [86], African Americans [87], Han Chinese [88,89]		
rs17487792	↑	Intronic	C>T			EA/US: high-risk [85]<br>Chinese: adrenal origin, stage IV [88]	European Americans [85], African Americans [87], Han Chinese [88]		
rs6712055	↑	Intronic	T>C			EA/US: high-risk [85]	European Americans [85], African Americans [87]		Breast – no risk [133]<br>Ovarian – increased risk [134]
rs7587476	↑	Intron 3	C>T			EA/US, AA/US: high-risk [85,87]	European Americans [85], African Americans [87]		
rs6715570	↑	Intronic	C>T			EA/US: high-risk [85]	European Americans [85]		

rs7585356	↓	3' UTR	G>A	Increases mRNA expression of BARD1-FL [86]		EA/US: high-risk, stage 4N, MYCN amp, age < 18 mos. [86]<br>Italians: stage 4N, age < 18 mos. [86]<br>Chinese: females [89]	European Americans [85,86], Italians [86], Han Chinese [89]	rs16852600 (intronic) [85,95]	
rs2070094	↓	Exon 6 (Val507Met)	G>A				European Americans [85], Italians [86]		Breast – benign [135-138], increased risk [139]
rs2229571	↑(US, Chinese) ↓(Italian)	Exon 4 (Arg378Ser)	G>C			Chinese: adrenal origin, stage IV [88]	European Americans [85], Italians [86], Han Chinese [88]	rs1048108 [88]	Breast – benign [135], decreased risk [136,137]<br>Cervical – benign [140]
rs1048108	↓	Exon 1 (Pro24Ser)	C>T [85,86]<br>G>A [92]	Negatively regulates cellular developme nt and modulates developme nt and apoptosis [95]		EA/US, Italians, AA/US: high-risk [92]	European Americans [85,92], Italians [86,92], African Americans, Spaniards [92]		Breast – benign [135], decreased risk [136,137]<br>Cervical – benign [140]
rs17489363	↑	5' UTR (promoter region)	C>T [92]<br>G>A [88]	Decreases mRNA expression of BARD1-FL [88,92]	Decreases transcriptional activity through binding of HSF1 [92]	EA/US, AA/US, Italians: high-risk [92]<br>Chinese: adrenal origin [88]	European Americans, African Americans, Italians, Spaniards [92], Han Chinese [88]	rs1129804<br>rs34732883 [88]	
rs16852804	↑	Intronic	C>T				African Americans [87]		
rs7599060	↑	Intronic	G>A			AA/US: high-risk [87]	African Americans [87]		
rs6720708	↑	Intronic	C>T			EA/US: adrenal origin [94]	European Americans [94]	rs17489363 [94]	

rs373888	↑	Exon 10 (Arg658Cys)	C>T			Chinese: adrenal origin [88]	Han Chinese [88]		Breast – benign or pathogenic [146-148] Ovarian – likely benign [19] Breast/Ovarian - likely benign [149,150]
rs3768707		Intronic	C>T [88] G>A [92]			Chinese: adrenal origin, stage III [88]	Han Chinese [88], European Americans [92]		
rs16852600		Intronic		Negatively regulates cellular developme nt and modulates developme nt and apoptosis [95]				rs7585356 [85]	Breast [135]
rs587781948		Exon 10	c.1921 C>T	Truncated protein p.Arg641X [115,116]					Breast – pathogenic [141-144]
p.Arg112X		Exon 3	c.334 C>T	Truncated protein p.Arg112X [115]					Breast – pathogenic [144,145]

Arrows indicate increased (↑) and decreased (↓) susceptibility to NB

Supplemental Table 2. All reported BARD1 variants in CRC, pancreatic cancer, NSCLC, nephroblastoma, Ewing sarcoma and AML

**Colorectal cancer**							
Reported as	Allelic change	Coding DNA location	Protein change	Function	Mechanism	Associates with	Presence in breast or gynecological cancers?
c.1811-2A>G	A>G	1811-2		Predicted to skip exon 9, leading to disruption of BRCT1 domain [105]		Strong CRC inheritance? [105]	
c.1217G>A p.Arg406Gln (rs587780014)	G>A	1217	Arg406Gln	Predicted to disrupt ANK and BRCT domains [106]	Inhibits apoptosis [106]	Stage III or IV CRC? Diagnosis at age less than 50? [106]	
c.1918C>A p.Leu640Ile (rs1553612535)	C>A	1918	Leu640Ile	Predicted to disrupt ANK and BRCT domains [106]	Inhibits apoptosis [106]	Stage III or IV CRC? Diagnosis at age less than 50? [106]	
β isoform [14]			Lacks exons 2 and 3 [14]			shorter survival [14]	Breast, ovarian, endometrial [13]
γ isoform [14]			Lacks exons 4-11 [14]		Stabilizes BARD1-FL [14]		Breast, ovarian, endometrial [13]
δ isoform [14]			Lacks exons 2-6 [14]			Age >60 y.o., increased survival [14]	Breast, ovarian, endometrial [13]
ε isoform [14]			Lacks exons 4-9 [14]				Breast, ovarian, endometrial [13]
η isoform			Lacks exons 2-9 [14]				Breast, ovarian, endometrial [13]
κ isoform			Lacks exon 3 [14]			T3 and T4, N1 and N2, Stage III and IV, shorter survival [14]	
π isoform			Lacks part of exon 4 [14]			Age >60 y.o., shorter survival [14]	
ϕ isoform			Lacks exons 3-6 [14]			Age >60 y.o., increased survival [14]	Breast, ovarian, endometrial [13]
BARD1-FL						Increased survival [14,101]	Breast, ovarian, endometrial [13]

1-7/9-11			Lacks exon 8 [101]				
1-2/4-11			Lacks exon 3 [101]				
1/4-11			Lacks exons 2-3 [101]				
1-7/10-11			Lacks exons 8-9 [101]				
1-6/10-11			Lacks exons 7-9 [101]				
1-4a/5-11			Lacks C-terminal region of exon 4 [101]				
1-3/5-11			Lacks exon 4 [101]				
1-2/5-11			Lacks exons 3-4 [101]				
1/5-11			Lacks exons 2-4 [101]				
1-3/5-7/10-11			Lacks exons 4, 8 and 9 [101]				
1/5-6/8-11			Lacks exons 2-4 and 7 [101]				
1/5-7/9-11			Lacks exons 2-4 and 8 [101]				
1-3/8-11			Lacks exons 4-7 [101]				
1-2/7-11			Lacks exons 3-6 [101]				
1/5-7/10-11			Lacks exons 2-4 and 8-9 [101]				
1/7-11			Lacks exons 2-6 [101]				
1-3/10-11			Lacks exons 4-9 [101]				
1-2/10-11			Lacks exons 3-9 [101]				
1/10-11			Lacks exons 2-9 [101]				

BARD1 9'L			Lacks exons 1-9 [103]	Encodes lncRNA composed of parts of exon 10 and 11 [103]	Inhibits miR-203 and -101 binding to 3' UTR, which reduce BARD1-FL and isoform mRNA expression [103]		
**Pancreatic cancer**							
Reported as	Allelic change	Coding DNA location	Protein change	Function	Mechanism	Associates with	
BARD1 c.632T> A p.Leu211*	T>A	632	Leu211X	Premature stop codon [113]		Familial PDAC? [113]	
BARD1 c.1921C>T p.Arg641X	C>T	1921	Arg641X	Premature stop codon [114]		PDAC? [114]	Breast – pathogenic [141-144]
BARD1 c.69_70delins25 p.Ala25Glyfs*41		69_70		Deletion-insertion → premature stop codon [119]		Pancreatico-duodenal NEN [119]	
rs2229571	G>C	1207	Arg378Ser [117]				Breast – benign [137], decreased risk [138-139]<br>Cervical – benign [142]
rs1129804	C>G	44	5' UTR [117]				
**Non-small cell lung cancer**							
Reported as	Allelic change	Coding DNA location	Protein change	Function	Mechanism	Associates with	
β isoform			Lacks exons 2 and 3 [18,122]	increased cellular proliferation, inhibited apoptosis and increased fibronectin expression [123]		Male sex, decreased DFS and OS [18]	Breast, ovarian, endometrial [13]

γ isoform			Lacks exons 4-11 [18,122]			Female sex [18]	Breast, ovarian, endometrial [13]
δ isoform			Lacks exons 2-6 [18,122]				Breast, ovarian, endometrial [13]
ε isoform			Lacks exons 4-9 [18,122]			Female sex [18]	Breast, ovarian, endometrial [13]
η isoform			Lacks exons 2-9 [18,122]			Female sex [18]	
κ isoform			Lacks exon 3 [18]			Male sex, decreased DFS and OS [18]	
π isoform			Lacks part of exon 4 [18]			Tumor progression, aggressive phenotype, decreased DFS and OS [18]	
ϕ isoform			Lacks exons 3-6 [18,122]				Breast, ovarian, endometrial [13]
BARD1-FL [18,122]							Breast, ovarian, endometrial [13]
BARD1 9'L			Lacks exons 1-9 [103]	Encodes lncRNA composed of parts of exon 10 and 11 [103]	Inhibits miR-203 and -101 binding to 3' UTR, which reduce BARD1-FL and isoform mRNA expression [103]		
**Nephroblastoma**							
Reported as	Allelic change	Coding DNA location	Protein change	Function	Mechanism	Associates with	
rs7585356	G>A					Increased susceptibility, Stage I and II [127]	
**Ewing sarcoma**							
Reported as	Allelic change	Coding DNA location	Protein change	Function	Mechanism	Associates with	
BARD1 c.176_177AG p.E59Afs*8		176_177	E59Afs*8	Frameshift mutation in RING domain [130]		Inheritance? [130]	

**Acute myeloid leukemia**							
Reported as	Allelic change	Coding DNA location	Protein change	Function	Mechanism	Associates with	
α isoform			Lacks exon 2 [17]				Breast, ovarian, endometrial [13]
β isoform			Lacks exons 2-3 [17]				Breast, ovarian, endometrial [13]
κ isoform			Lacks exon 3 [17]				
π isoform			Lacks part of exon 4 [17]				
ω1 isoform			Lacks exons 1 through N-terminus of exon 4 [17]	Defective mitosis, inhibits apoptosis [17]	Sequesters and stabilizes p53 [17]		Breast, ovarian, endometrial, cervical – alternative start site [13]
